# Intracellular acidification is required for full activation of the sweet taste receptor by miraculin

**DOI:** 10.1038/srep22807

**Published:** 2016-03-10

**Authors:** Keisuke Sanematsu, Masayuki Kitagawa, Ryusuke Yoshida, Satoru Nirasawa, Noriatsu Shigemura, Yuzo Ninomiya

**Affiliations:** 1 Section of Oral Neuroscience, Graduate School of Dental Science, Kyushu University, 3-1-1 Maidashi, Higashi-ku, Fukuoka 812-8582, Japan; 2 OBT Research Center, Faculty of Dental Science, Kyushu University, 3-1-1 Maidashi, Higashi-ku, Fukuoka 812-8582, Japan; 3 Japan International Research Center for Agricultural Sciences, 1-1 Ohwashi, Tsukuba, Ibaraki 305-8686, Japan; 4 Division of Sensory Physiology, Research and Development Center for Taste and Odor sensing, Kyushu University, 3-1-1 Maidashi, Higashi-ku, Fukuoka 812-8582, Japan

## Abstract

Acidification of the glycoprotein, miraculin (MCL), induces sweet taste in humans, but not in mice. The sweet taste induced by MCL is more intense when acidification occurs with weak acids as opposed to strong acids. MCL interacts with the human sweet receptor subunit hTAS1R2, but the mechanisms by which the acidification of MCL activates the sweet taste receptor remain largely unexplored. The work reported here speaks directly to this activation by utilizing a sweet receptor TAS1R2 + TAS1R3 assay. In accordance with previous data, MCL-applied cells displayed a pH dependence with citric acid (weak acid) being right shifted to that with hydrochloric acid (strong acid). When histidine residues in both the intracellular and extracellular region of hTAS1R2 were exchanged for alanine, taste-modifying effect of MCL was reduced or abolished. Stronger intracellular acidification of HEK293 cells was induced by citric acid than by HCl and taste-modifying effect of MCL was proportional to intracellular pH regardless of types of acids. These results suggest that intracellular acidity is required for full activation of the sweet taste receptor by MCL.

One of the most puzzling and striking phenomena in human taste perception is the induction of a strong sweet taste by a sour acid when the sour acid is preceded by tasting a plant-based glycoprotein (itself having no taste) called miraculin (MCL)^1^,^2^. This 191 amino acid glycoprotein forms a homodimeric complex. Taste-modifying effect of MCL is specific to humans but not to rodents^2^,^3^,^4^,^5^.

Sweet taste is mediated by the heterodimer receptor of taste type 1 receptor proteins, TAS1R2 and TAS1R3, expressed in subsets of taste receptor cells^6^,^7^. The TAS1Rs belong to the class C G-protein-coupled receptor (GPCR) family, and consist of three principal domains: an amino-terminal domain (ATD) and a cysteine-rich domain (CRD) located in the extracellular region, and a transmembrane domain (TMD)^8^. A previous study suggested that amino acid residues 448 to 494 in the ATD of hTAS1R2 are required for sweet taste induction by MCL^9^. Replacement of histidine residues in MCL with alanine leads to the loss of this effect suggesting that acidification induces a necessary conformational change in MCL^10^.

Psychophysical studies have shown that the type of acid affects the strength of the taste modifying effect of MCL: Weak acids produce a more potent taste-modifying effect than do strong acids^2^. This difference is also observed in sour taste alone as sour sensation is more intense with weak acids than strong acids^2^. Here we provide a molecular explanation for this remarkable phenomenon and argue that it provides new insights into the fundamental mechanisms underlying sour taste perception.

## Results

### Taste-modifying effect of MCL requires the ATD of hTAS1R2

**Figure 1:**
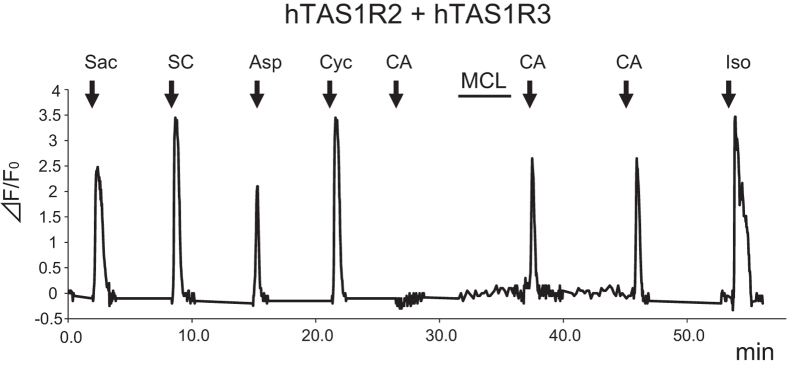
The taste-modifying compound miraculin (MCL) induces its effect via the human sweet taste receptor. Typical example of single cell Ca^2+^ imaging is shown. HEK293 cells heterologously expressing hTAS1R2 + hTAS1R3 showed calcium responses to non-nutritive sweeteners 10 mM saccharin (Sac), 0.3 mM SC45647 (SC), 10 mM aspartame (Asp) and 30 mM cyclamate (Cyc), but not to 3 mM citric acid (CA: pH 5.0). After application of MCL (10 μg/ml), this cell showed responses to citric acid. Control: 10 μM isoproterenol (Iso).

**Figure 2:**
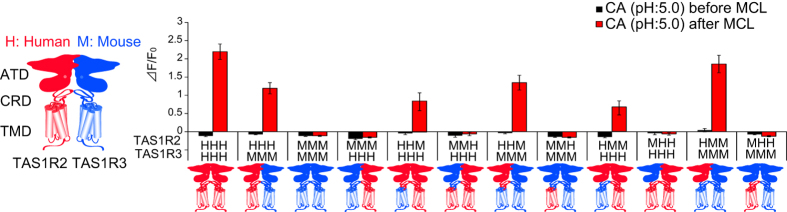
The amino-terminal domain (ATD) of hTAS1R2 is required for sensitivity to MCL. HEK293 cells were transiently transfected with full length and/or chimera of TAS1R2 and TAS1R3, and Gα16-gust44. TAS1R2 (*left*) and TAS1R3 (*right*) are shown by schematic receptors. ATD, amino-terminal domain; CRD, cysteine-rich domain; TMD, transmembrane domain. The full length and the chimera of TAS1Rs are indicated by three letters including of *H* (human; *red*) or *M* (mouse; *blue*) (example, chimera containing the ATD and the CRD of human receptor coupled to the TMD of mouse receptor as for HHM). The responses of the receptors to 3 mM citric acid (CA: pH 5.0) before and after application of MCL (10 μg/ml) were examined. 15–30 cells. Data are expressed as means ± S.E.

We examined the effect of solution properties (e.g., pH) on the interaction of MCL with the sweet taste receptor using transient transfection of HEK293 cells by the sweet taste receptor, TAS1R2 + TAS1R3, along with Gα16-gust44. Receptor activity was monitored by calcium responses. We found that these cells were sensitive to various sweet compounds, but not to citric acid (pH 5.0). Upon application of MCL (10 μg/ml) alone at neutral pH, the transfected cells showed no calcium response. However, when citric acid was subsequently added, a calcium response was observed consistent with the sweet-inducing effect of MCL (Fig. 1). This effect was not observed in HEK293 cells expressing mouse mTas1r2 + mTas1r3 (Fig. 2). These results are also consistent with the species-specific effect of MCL as previously shown by psychophysical and electrophysiological studies^2^,^3^,^4^,^5^.

To identify portions of hTAS1R2 and hT1ASR3 required for the sensitivity to MCL, we examined responses of combinations of full length or chimera receptors in human and/or mouse. First, we confirmed that these receptors were functional, except for a heterodimer mTas1r2 + hTAS1R3 as described previously (Supplementary Fig. S1)^11^. These receptors showed responses to artificial sweeteners SC45647 and saccharin. Heterodimers containing the ATD of hTAS1R2 and the TMD of hTAS1R3 showed sensitivity to artificial sweeteners aspartame and cyclamate, consistent with previous studies on binding sites^12^,^13^,^14^ (Supplementary Fig. S1). Second, responses to citric acid (pH 5.0) after application of MCL did not depend on whether TAS1R3 originated from mouse or human (Fig. 2). The heterodimer hTAS1R2 + mTas1r3 responded to MCL, showing the species specificity of TAS1R2 but not TAS1R3. Third, the human ATD of TAS1R2 was necessary for the response since heterodimers containing TAS1R2HHM (chimera containing ATD and CRD of human receptor coupled to the TMD of mouse receptor as for HHM) and HMM but not MMH and MHH chimeras showed responses (Fig. 2). From these results, we conclude that the ATD of hTAS1R2 is required for the sweet-inducing effect of MCL.

### Intracellular acidification enhances taste-modifying effect of MCL

**Figure 3:**
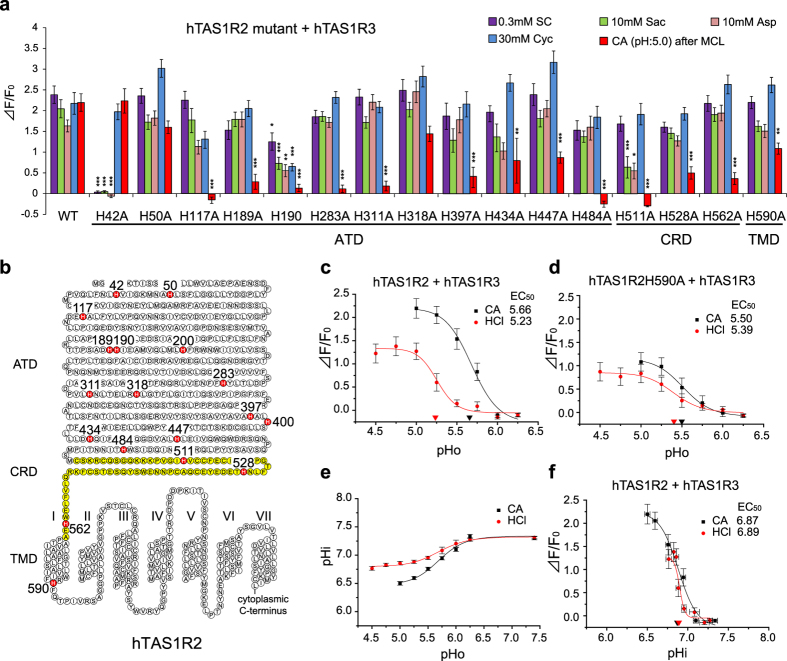
Histidine residues in hTAS1R2 are required for taste-modifying effect of MCL. (**a**) HEK293 cells were transiently transfected with hTAS1R2 (WT or mutant), hTAS1R3, and Gα16-gust44. The responses of the receptors to 0.3 mM SC45647 (SC), 10 mM saccharin (Sac), 10 mM aspartame (Asp), 30 mM cyclamate (Cyc) and 3 mM citric acid (CA: pH 5.0) after application of MCL (10 μg/ml) were examined. 15–30 cells. Responses to test solutions were analyzed by multivariate ANOVA followed by Tukey-Kramer test. *P < 0.05, **P < 0.01, ***P < 0.001 *vs. WT*. (**b**) A snake plot of hTAS1R2. Histidine residues in hTAS1R2 are indicated in red. ATD, Amino-terminal domain; CRD, cysteine-rich domain (shown in yellow); TMD, transmembrane domain. (**c**) HEK293 cells were transiently transfected with hTAS1R2, hTAS1R3, and Gα16-gust44. The extracellular pH (pHo) dependent responses of the receptors to 3 mM citric acid (CA) and 7 mM HCl after application of MCL (10 μg/ml) were examined. 18 cells. (**d**) HEK293 cells were transiently transfected with hTAS1R2H590A, hTAS1R3 and Gα16-gust44. The pHo-dependent responses of the receptors to 3 mM CA and 7 mM HCl after application of MCL (10 μg/ml) were examined. 15–19 cells. (**e**) Intracellular pH (pHi) of HEK293 cells was measured after application of different pH (pHo) of 3 mM CA and 7 mM HCl. 145–148 cells. (**f**) Intracellular pH response curves were obtained by using the same solutions of 3 mM citric acid and 7 mM HCl in (**c**). 18 cells. Values are means ± S.E.

Previous studies showed that replacement of histidine residues to alanine in MCL and another taste modifier neoculin reduced their taste-modifying effects, suggesting that protonation of histidine residues in MCL and neoculin is important for the acid-induced sweetness^10^,^15^,^16^. Protonation of histidine residues would also occur in the sweet taste receptor. Therefore, we focused on the histidine residues of hTAS1R2 because protonation of these histidine residues may also affect the receptor binding and activity of MCL. We looked at the effect of alanine replacement in hTAS1R2 on responses to several sweeteners as well as 3 mM citric acid after application of MCL (Fig. 3a,b). These responses were significantly different among different mutants (Supplementary Table S1). We found that hTAS1R2H42A showed no response to SC45647, saccharin and aspartame and that hTAS1R2H190A and H511A showed smaller responses to various sweet compounds than wild-type (WT) (post-hoc Tukey-Kramer test, Ps < 0.05 vs. WT, SC45647, saccharin, aspartame, and cyclamate for H190A; saccharin, and aspartame for H511A) (Fig. 3a). Responses to various sweet compounds in other mutants were not significantly different from those in WT (post-hoc Tukey-Kramer test, Ps > 0.05 vs. WT). Concerning the effect of MCL, alanine replacements of His-117, 189, 190, 283, 311, 397, and 484 in the ATD, and His-511, 528, and 562 in the CRD of hTAS1R2 abolished or greatly reduced the receptor’s sensitivity to citric acid after MCL to below 30% of WT (post-hoc Tukey-Kramer test, Ps < 0.001 vs. WT). Mutations of His-434 and 447 in the ATD and His-590 in the TMD moderately reduced the sweet-inducing effect of MCL to 30–70% of WT (post-hoc Tukey-Kramer test, Ps < 0.01 vs. WT). These results indicate that mutating histidine for alanine in the ATD of TAS1R2 yields a receptor unable to respond to citric acid after application of MCL.

The effect of MCL is known to differ among different classes of acids in psychophysical and electrophysiological studies in humans and rhesus monkeys^2^,^3^. This was tested in our expression system using citric acid (a weak acid) and HCl (a strong acid) (Fig. 3c). We found significant differences in responses to citric acid and HCl after MCL (Fig. 3c and Supplementary Table S2), indicating that weak acids are more potent than strong acids at inducing the taste-modifying effect of MCL.

In mammals the sensation of sourness correlates with titratable acidity better than it does with pH^17^. Indeed, many studies have reported that intracellular pH is key to the sensation of “sourness”^18^,^19^,^20^,^21^. For instance, at the same pH, citric acid induces greater decrease in intracellular pH than HCl by penetrating plasma membrane as undissociated molecules^18^ and is a more intense sour stimulus than HCl^18^,^19^,^20^,^21^. The taste-modifying effect of MCL by acids exhibits similar properties^2^. Therefore, variations in intracellular acidification may underlie this difference and we tested whether mutation of histidine residues in the intracellular region of hTAS1R2 (hTAS1R2H590A) affects the modifying ability of MCL (Fig. 3b,d). As expected, the difference in response to citric acid and HCl after MCL was abolished in this mutant (Supplementary Table S2), although responses themselves became smaller compared to those of WT (Supplementary Table S3). Responses to various sweet compounds were not significantly different between this mutant and WT (Fig. 3a), suggesting that this mutation probably does not affect the receptor activation system.

In addition, we monitored intracellular pH (pHi) in HEK293 cells stimulated with weak and strong acids at different extracellular pH (pHo) (Fig. 3e and Supplementary Table S4) and examined the relationship between sweet-inducing effect of MCL and pHi (Fig. 3f). Undissociated acids, mainly weak acids, can enter into the intracellular region through the cell membrane, inducing acidification intracellularly. Heightened intracellular acidification was induced by citric acid compared with HCl (Fig. 3e and Supplementary Table S4). At the same pHi, the calcium responses to citric acid and HCl after MCL were essentially the same (Fig. 3f). The pHi-dependent curve of citric acid was close to that of HCl (Fig. 3f), indicating that sweet-inducing effect of MCL is mainly proportional to pHi.

Bathing cells in buffer containing NaHCO_3_ and sodium acetate at neutral pH induces intracellular acidification without changing extracellular pH^22^. We also tested whether such procedures inducing selective intracellular acidification (pHi: ~6.8 and pHo: 7.2) fully activate the miraculin-bound receptor. After application of MCL, HEK293 cells exhibited no response to these solutions (Supplementary Fig. S2), indicating that both extracellular and intracellular acidification are required for full activation of the sweet taste receptor by MCL.

## Discussion

MCL induces taste-modifying effects in humans, chimpanzees and rhesus monkeys but not in rodents^2^,^4^,^5^,^23^. In accordance with those psychophysical and electrophysiological studies, MCL interacted with the human, but not mouse sweet receptor in our sweet receptor assay (Fig. 2), indicating the species-specific effect of MCL. A previous study suggested that the interaction site for MCL is the ATD of hTAS1R2 and the 448 to 494 amino acid residues in hTAS1R2 are required for the effect^9^. We also demonstrated that the ATD of hTAS1R2 is required for sweet-inducing effect of MCL (Fig. 2).

Mutation of His-42 to alanine in hTAS1R2 abolished sensitivity to aspartame, saccharin, and SC45647 but not to cyclamate (Fig. 3a). His-42 is located in the entrance of the orthosteric binding site for aspartame and saccharin in the ATD of hTAS1R2^13^. Indeed, Ser-40, close to His-42, is known to be one of the critical residues for the species-dependent difference in sensitivity to aspartame^24^. Our results suggest that replacement of His-42 with alanine affects conformation of the binding site for aspartame, saccharin, and SC45647 in hTAS1R2 but not the binding site for MCL in hTAS1R2 and cyclamate in hTAS1R3. Thus receptor activation through the interaction between hTAS1R2 and MCL may not require an activation system used by low-molecular weight sweet compounds (aspartame, saccharin, and SC45647) in the ATD of hTAS1R2.

Following replacement of histidine residues to alanine in the extracellular region of hTAS1R2, the taste-modifying effect of MCL was abolished (hTAS1R2-H117A, H484A, H511A) or reduced (hTAS1R2-H189A, H190A, H283A, H311A, H397A, H434A, H447A, H528, and H562A) (Fig. 3a). In some mutants (hTAS1R2H190A and H511A), responses to sweet compounds were significantly reduced (Fig. 3a), suggesting that these mutants have a severe impairment of their receptor activation system. In the other mutants with abolished or reduced MCL effects, mutations may not disturb the receptor activation system since responses to several sweet compounds were not significantly affected (Fig. 3a). We speculate that the binding affinity of MCL to the sweet receptor may not be altered in mutations reducing of the effect of MCL, because the sweet-inducing effect of MCL lasted at least 10 min despite the continuous washing out of the cell surface. We hypothesize that these mutations may abolish or reduce proton-induced interaction between the sweet receptor and MCL, which leads to activation of the sweet receptor. Also it is possible that binding affinity of MCL to the sweet receptor may be affected by these mutations in TAS1R2. These possibilities should be tested in future studies.

The effectiveness of MCL is known to differ among acids. In humans, the order of the effectiveness of MCL to induce the sweetness by each of acids is as follows: acetic acid (pKa: 4.76), formic acid (pKa: 3.75), lactic acid (pKa: 3.86), oxalic acid (pKa: 1.27) and HCl (pKa: −8.0)^2^. In our assay citric acid (weak acid) also induced stronger response than HCl (strong acid) at the same pH (Fig. 3c). Weak acid produces more undissociated form of acid than strong acid. Undissociated form of acid could enter into the intracellular region through the cell membrane, which induces acidification intracellularly. Thus weak acids are able to induce intracellular acidification more strongly than strong acids and we demonstrated this using HEK cells (Fig. 3c). Intracellular pH was more closely associated with MCL activity than extracellular pH (Fig. 3c,f). These results strongly suggest that intracellular acidification could affect the taste-modifying effect of MCL.

**Figure 4:**
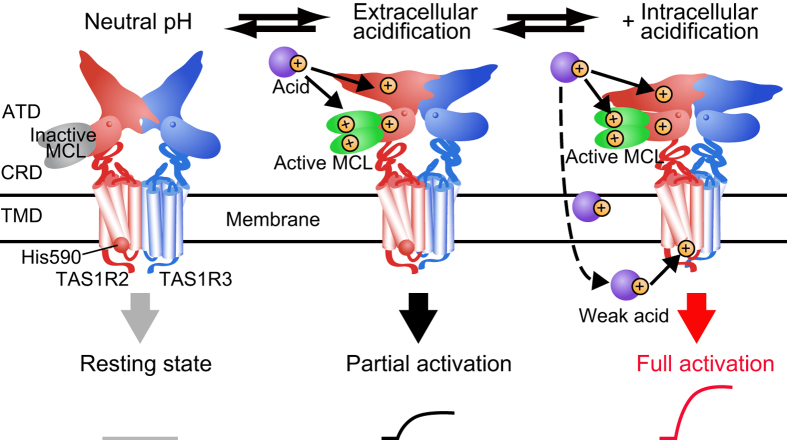
Proposed model for the taste-modifying effect of MCL. MCL (inactive form) binds with the amino-terminal domain of hTAS1R2 at neutral pH, which does not induce activation (left). Extracellular acidification induces partial activation of sweet receptor through the interaction between extracellularly protonated hTAS1R2 and active form of MCL (center). In addition, weak acid enters into intracellular region through cell membrane and causes intracellular acidification leading to full activation.

Based on our results, we propose a model underlying the taste-modifying effect of MCL (Fig. 4). MCL binds with the ATD of hTAS1R2 as an inactive form at neutral pH. After sufficient extracellular acidification, the extracellular region of hTAS1R2 and MCL are protonated, leading to activation of an intracellular signalling cascades. When a weak acid is applied, undissociated acids cross the membrane of the taste receptor cell, protonating the interior to a level dependent upon the strength of acid. This intracellular acidification leads to a protonation of the intracellular domain of hTAS1R2. Then, full receptor activation is evoked by the interaction between the fully protonated hTAS1R2 and active MCL.

## Methods

All experimental procedures were performed in accordance with the National Institutes of Health Guide for the Care and Use of Laboratory Animals and approved by the committee for Laboratory Animal Care and Use at Kyushu University, Japan.

### Preparation of chimeras and point mutations

Human TAS1Rs and Gαl6-gust44 expression constructs were generated in the pEF-DEST51 Gateway vector (Life Technologies Corporation)^11^,^25^. Mouse Tas1r2 and Tas1r3 were cloned as described^26^,^27^. Construction of human/mouse chimeras of TAS1Rs was performed by PCR using overlapping primers^28^. Point mutations in TAS1R clones were made by site-directed mutagenesis (TAKARA). To subclone each gene into the vector, a Kozak cassette was introduced at the 5′ end before the start codon. The integrity of all DNA constructs was confirmed by DNA sequencing. There are 18 histidine residues in hTAS1R2. Sixteen mutants replacing histidine in hTAS1R2 with alanine were created and their functions were examined. One mutant (hTAS1R2H400A) could not be obtained and the other one (hTAS1R2H200A) did not make a functional receptor.

### Functional expression

As described previously^11^, HEK293 cells were kindly provided by Dr. Makoto Tominaga (Okazaki Institute for Integrative Bioscience). These cells were cultured at 37 °C under a humidified atmosphere containing 5% CO_2_ in Dulbecco’s modified Eagle’s medium supplemented with 10% fetal bovine serum. To obtain reproducible Ca^2+^ responses, cells were split every 2 days before the cells became confluent. Cells were discarded after 2 months of passages and new cells were prepared from frozen-stock. For calcium imaging experiments, cells were seeded onto a 35 mm recording chamber. After 24 hrs at 37 °C, confluent cells (60–70%) were washed in OptiMEM medium supplemented with GlutaMAX-I (Life Technologies Corporation) and plasmid DNAs were transiently cotransfected into HEK293 cells using Lipofectamine2000 regent (Life Technologies Corporation) (2.0 μl per 1.0 μg DNA). TAS1Rs (or their mutants) and Gα16-gust44 were transfected using 0.3 μg of plasmids for 35 mm recording chambers. Ca^2+^ imaging assays were performed 24 hrs after transfection.

### Single cell Ca^2+^ imaging

As described previously^11^, a bath perfusion system was used for determination of the kinetics of activation. Transfected cells in 35 mm recording chambers were washed in Hank’s balanced salt solution (HBSS) (Life Technologies Corporation) containing 10 mM HEPES (pH 7.4), and loaded with 3.0 mM Fluo-4 acetoxymethyl ester (Life Technologies Corporation) for 30 min at 37 °C. The dye-loaded cells were subjected to Ca^2+^ imaging. Taste solutions diluted in HBSS containing 10 mM HEPES were applied sequentially to the cells for 30 s with a peristaltic pump at a flow rate of 1.5 ml/min, and fluorescence images were obtained using a S Fluor 620/0.75 objective lens (Nikon) via a cooled-CCD camera (C6790, Hamamatsu Photonics) fitted to a TE300 microscope (Nikon). AquaCosmos software (v. 1.3, Hamamatsu Photonics) was used to acquire and analyze fluorescence images. A 5 min interval was maintained between each tastant application to ensure that the cells were not desensitized as a result of the previous application of taste solutions. Responses were measured from individual responding cells. MCL (10 μg/ml) were applied to the HEK293 cells for 3 min. Isoproterenol (Iso, 10 μM) was used as positive control, which stimulates endogenous β-adrenergic receptors, providing that the Gα16-dependent signal transduction cascade was functional.

### Solutions

Chemical compounds were disolved in HBSS containing 10 mM HEPES. Chemical compounds used in this study were saccharin (10 mM), SC45647 (0.3 mM), aspartame (10 mM), and sodium cyclamate (30 mM) as sweet taste stimuli and isoproterenol (10 μM) as positive control. We used citric acid (pK_a_: 3.09) (concentration 3 mM in HBSS containing 10 mM HEPES, pH 7.4, 6.25, 6.0, 5.75, 5.5, 5.25, 5.0 adjusted using NaOH), and HCl (pKa: −8.0) (concentration 7 mM in HBSS containing 10 mM HEPES, pH 7.4, 6.25, 6.0, 5.75, 5.5, 5.25, 5.0, 4.75, 4.5 adjusted using NaOH), as a weak acid and strong acid, respectively. We did not use weaker acid like acetic acid (pKa: 4.76) than citric acid, because our pilot studies showed that acetic acid frequently induced nonspecific calcium responses in HEK293 cells in the absence of transfected receptor DNAs as reported previously^29^. For the same reason, citric acid was limited in its usable pH (pH 5.0 or above). In all the experiments, we confirmed that acid solutions did not produce calcium responses before application of MCL by means of single cell Ca^2+^ imaging. For the experiment of intracellular acidification with extracellular neutral pH, we used modified HBSS; 90 mM bicarbonate solution (in mM) 1.26 CaCl_2_, 0.493 MgCl_2_, 0.407 MgSO_4_ 5.33 KCl 0.441 KH_2_PO_4_ 90 NaHCO_3_ 48 NaCl 0.338 Na_2_HPO_4_ 10 HEPES and 27.5 mM acetate solution (in mM) 1.26 CaCl_2_, 0.493 MgCl_2_, 0.407 MgSO_4_ 5.33 KCl 0.441 KH_2_PO_4_ 4.17 NaHCO_3_ 110 NaCl 0.338 Na_2_HPO_4_ 27.5 CH_3_COONa 10 HEPES and pH were adjusted to 7.2 by titration with HCl^22^. For the calibration of intracellular pH using SNARF, we used modified HBSS (in mM): 1.26 CaCl_2_, 0.493 MgCl_2_, 0.407 MgSO_4_ 140 KCl 0.441 KH_2_PO_4_ 4.17 NaHCO_3_ 0.338 Na_2_HPO_4_ 10 HEPES: with 10 μM nigericin and pH was adjusted to 7.6, 7.4, 7.2, 7.0, 6.8, 6.6, 6.4, 6.2 with NaOH or HCl. Regents were purchased from Nacalai tesque (Isoproterenol), Ajinomoto (Aspartame) and Wako Pure Chemical Industries (others). MCL was purified from pulps of the miracle fruits (*Richadella dulcifica*) as reported previously^29^.

### Intracellular pH imaging

HEK 293 cells were loaded with SNARF-5F-acetoxymethyl ester (Life Technologies Corporation) for 30 min at 37 °C. The same perfusion system in Ca^2+^ imaging was used to apply acid solutions. SNARF dye was excited with 488 nm laser in NIS elements (Nikon), and the ratio of fluorescence emissions at 640 and 580 nm (F640/F580) were captured every 5 s using a Nikon C2Si camera and Image Suite software (Nikon). Intracellular pH were measured by calibration curve obtained from modified HBSS at different pH^30^,^31^.

### Data Analysis

In the analysis of single cell responses, changes in [Ca^2+^]_i_ were monitored as changes in fluo-4 fluorescence. Fluorometric signals are expressed as relative fluorescence changes: ΔF/F_0_ = (F–F_0_)/F_0_, where F_0_ denotes the baseline fluorescence level. The magnitude of the calcium increases from 10 to 30 s after stimulus onset were measured and averaged. The data were expressed as the mean ± S.E. of the ΔF/F_0_ value and used for statistical analysis. EC_50_ values were calculated from individual cumulative pH-dependent-response data using curving-fitting routines of Origin 5.0 (Microcal Software). The effects of acid solution and mutation were analyzed by a two-way ANOVA and the post hoc Tukey-Kramer test. The effects of mutation on sweet responses were evaluated by multivariate ANOVA and the post-hoc Tukey-Kramer test. All calculations were performed using the statistical software package IBM SPSS Statistics (IBM).

## Additional Information

**How to cite this article**: Sanematsu, K. *et al.* Intracellular acidification is required for full activation of the sweet taste receptor by miraculin. *Sci. Rep.* **6**, 22807; doi: 10.1038/srep22807 (2016).

## Supplementary Material

Supplementary Information
