# Supplementary material for: Intracellular acidification is required for full activation of the sweet taste receptor by miraculin
Source: Sci Rep. 2016 Mar 10;6:22807. doi: 10.1038/srep22807 (PMC4785348; doi:10.1038/srep22807)
Supplement: Supplementary Information [file srep22807-s1.pdf]

Supplementary Information for:

Intracellular acidification is required for full activation of sweet taste receptor by miraculin

Keisuke Sanematsu[1], Masayuki Kitagawa[1], Ryusuke Yoshida[1,2], Satoru Nirasawa[3], Noriatsu Shigemura[1], and Yuzo Ninomiya[1,4]*

Author affiliation:

[1]Section of Oral Neuroscience, Graduate School of Dental Science, [2]OBT Research Center, Faculty of Dental Science, and [4]Division of Sensory Physiology, Research and Development Center for Taste and Odor sensing, Kyushu University, 3-1-1 Maidashi, Higashi-ku, Fukuoka 812-8582, Japan

[2]Japan International Research Center for Agricultural Sciences, 1-1 Ohwashi, Tsukuba, Ibaraki 305-8686, Japan

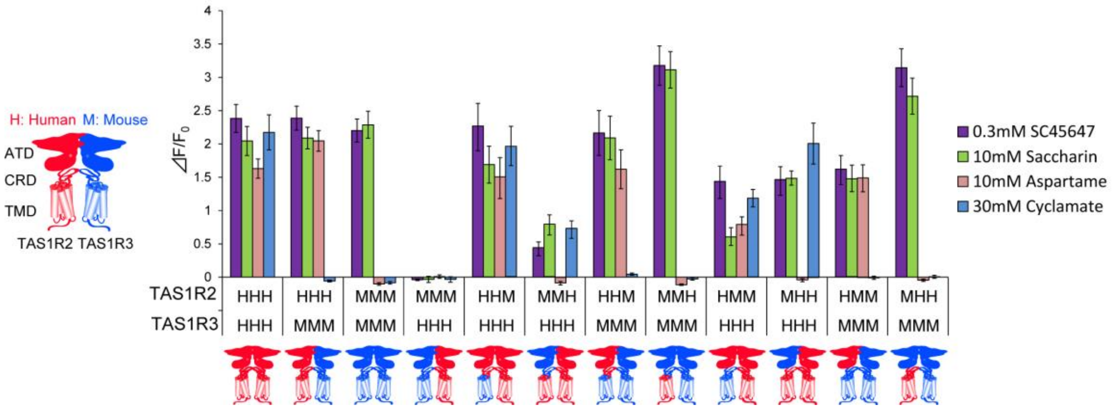

Supplementary Figure S1: Responses to sweet substances in HEK293 cells expressing combinations of full length or chimera receptors in human and/or mouse. HEK293 cells were transiently transfected with full length or chimera of TAS1R2 and TAS1R3 and Gα16-gust44. TAS1R2 (left) and TAS1R3 (right) are shown by schematic receptors. The full lengths and the chimeras of TAS1Rs are indicated by three letters including of *H* (human; *red*) or *M* (mouse; *blue*) (example, chimera containing the ATD and the CRD of human receptor coupled to the TMD of mouse receptor as for HHM). The responses of the receptors to 0.3 mM SC45647, 10 mM saccharin, 10 mM aspartame and 30 mM cyclamate were examined. Values are means ± S.E. of 15–30 cells.

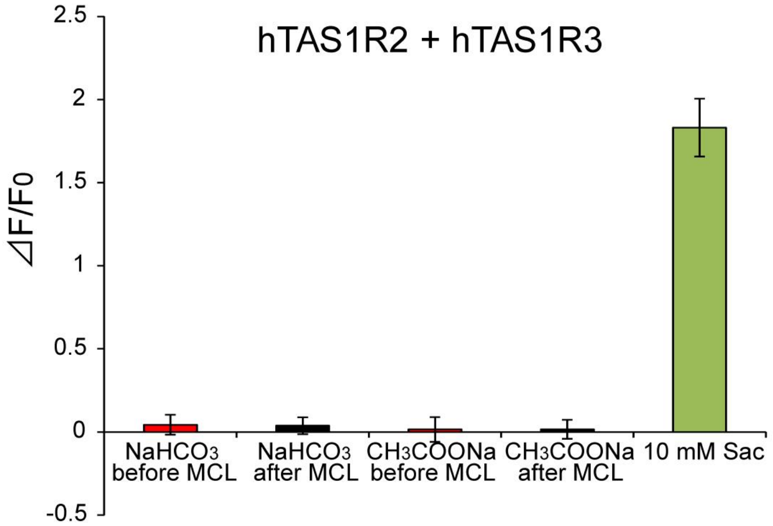

Supplementary Figure S2: Intracellular acidification with extracellular neutral pH does not induce taste-modifying effect of miraculin. HEK293 cells were transiently transfected with hTAS1R2, hTAS1R3 and Gα16-gust44. The responses of the receptors to $NaHCO_3$ (90 mM, pHi: ~6.8, pHo: 7.2) and sodium acetate (27.5 mM, pHi: ~6.8, pHo: 7.2) before and after application of miraculin (10 μg/ml) were examined. Response to 10 mM Saccharin (Sac) was examined as positive control. Values are means ± S.E. of 20 cells.

Supplementary Table S1. **Results for multivariate ANOVA of calcium responses to sweet compounds.** The effects of mutagenesis to responses to various sweet compounds (0.3 mM SC45647, 10 mM saccharin, 10 mM aspartame, 30 mM cyclamate) and 3 mM citric acid after miraculin (MCL) were analyzed by multivariate ANOVA. The table is based on data shown in Fig. 3a. ***$P < 0.001$

	Degrees of freedom	*F* value
SC45647	16, 302	8.94***
saccharin	16, 302	8.38***
aspartame	16, 302	12.6***
cyclamate	16, 302	9.35***
citric acid after MCL	16, 302	22.16***

Supplementary Table S2. **Results for two-way ANOVA of calcium responses to acids after MCL in hTAS1R2/hTAS1R3 and hTAS1R2H590A/hTAS1R3 (citric acid vs. HCl).** The effect of acid solution were analyzed by two-way ANOVA. The table is based on data shown in Fig. 3c, d. ***P < 0.001.

	hTAS1R2/hTAS1R3		hTAS1R2H590A/hTAS1R3	
	Degrees of freedom	*F* value	Degrees of freedom	*F* value
acid solution	1, 204	59.61***	1, 216	3.19
acid solution × concentration	5, 204	9.43***	5, 216	0.83

Supplementary Table S3. **Results for two-way ANOVA of calcium responses to citric acid and HCl after MCL (hTAS1R2/hTAS1R3 vs. hTAS1R2H590A/hTAS1R3).** The effect of genotype were analyzed by two-way ANOVA. The table is based on data shown in Fig. 3c, d. * $P < 0.05$, ***$P < 0.001$.

	Citric acid		HCl	
	Degrees of freedom	*F* value	Degrees of freedom	*F* value
genotype	1, 210	32.99***	1, 272	6.91**
genotype × concentration	5, 210	5.05***	7, 272	2.17*

Supplementary Table S4. **Results for two-way ANOVA of intracellular pH after application of acids (citric acid vs. HCl).** The effect of acid solution were analyzed by two-way ANOVA. The table is based on data shown in Fig. 3e. ***P < 0.001.

	Degrees of freedom	*F* value
acid solution	1, 291	19.16***
acid solution × concentration	6, 1746	33.34***
